# Are we all in this together?: mathematics teachers’ perspectives on equity in remote instruction during pandemic

**DOI:** 10.1007/s10649-021-10060-1

**Published:** 2021-06-02

**Authors:** Zuhal Yılmaz, Hande Gülbağcı Dede, Ruthmae Sears, Selin Yıldız Nielsen

**Affiliations:** 1 Graduate School of Education, University of California Riverside, 1207 Sproul Hall, Riverside, CA 92521 USA; 2 Department of Mathematics and Science Education, Marmara University, Istanbul, Turkey; 3 Department of Teaching and Learning College of Education, University of South Florida, Tampa, USA; 4 Global Studies Department, University of California Riverside, Riverside, CA USA

**Keywords:** Accessibility, COVID-19, Equitable mathematics instruction, Language barrier, Socioeconomic status

## Abstract

The COVID-19 pandemic is an unprecedented situation that influenced all aspects of society, including education. Millions of students found themselves adjusting to a new medium of mathematics instruction, not to mention the teachers who had to provide instruction through remote sources. Considering students’ diverse social, economic, and academic background, this study sought to examine teachers’ perspectives on factors that support or hinder how equity is attended to in mathematics during remote instruction and the extent it differed from practices utilized when instruction was provided in a face-to-face setting. We also sought to document teachers’ perspectives on how they attended to equity in mathematics to support students with language barriers. We interviewed nine teachers to explore their perspectives of factors that support or hinder equity in mathematics teaching and learning during remote instruction compared to face-to-face instruction and how they support the diverse needs (inclusive of language barriers) of students. There were salient factors in this study that supported or hindered equitable mathematics instruction, such as teachers’ beliefs, expectations for students, access to resources, students’ socioeconomic status, and language barriers. Hence, it is recommended that policymakers, school administrators, and teachers need to collaborate to systematically plan to ensure that all students have access to quality mathematics.

## Introduction

The coronavirus disease 2019 (COVID-19) caused a pandemic that disrupted the normalcy of educational institutions (Bakker & Wagner, 2020). Many educational entities, in approximately 190 countries, transitioned from face-to-face to remote modes of instruction to prevent the spread of the disease (UNESCO, 2020). This paradigm shift placed a tremendous strain on educational communities and policymakers to utilize technology to support all aspects of the mathematics curriculum (Remillard & Heck, 2014) and to identify measures that could be utilized to assess learning. The demand to provide remote instruction also amplified access and equity issues relative to the availability of technological tools and infrastructure within local communities and factors that may impact teachers’ adoption and integration of information and communication technologies (Buabeng-Andoh, 2012; Warschauer & Matuchniak, 2010). Different countries addressed educational demands in different ways depending on their technological and accessibility capabilities. In Turkey, lecture videos broadcasted on television and synchronous online instruction were used to provide access to mathematics during remote instruction.

### Nationalized remote instruction: Turkey context-COVID-19

Turkey has a centralized educational system with a nationalized curriculum and compulsory formal education for grades 1–12, since 2012 (Gökmenoğlu & Clark, 2015). The mathematics curriculum envisions a student-centered conceptual understanding of mathematics teaching and learning (Ministry of National Education (MoNE), 2018a, b). Remote mathematics instruction has been carried out within two mediums: synchronous lessons integrated into an Education Information Network (EBA, https://www.eba.gov.tr/) or other online platforms and the EBA-TV^1^.

There are challenges in attending to equity during synchronous mathematics lessons, due to limited access to the Internet, particularly for students with low socioeconomic status (Aktaş Salman, 2020; OECD, 2020). There is also a gap between individuals’ access to the Internet from the east and west regions of Turkey (Turkish Statistical Institute (TUIK), 2019b). It was reported that 49.1% of the population accessed fixed broadband Internet and 97.7% accessed television (TUIK, 2019a). Thus, 8GB Internet per month was provided through smartphones to enable equal access to synchronous lessons (MoNE, 2020), which may not be enough for accessing all lessons. Educational content is also broadcast through EBA-TV as a second medium.

The freely accessible state channel (TRT) provides infrastructure for educational contents in EBA-TV that broadcasts mathematics lecture videos that follow the mathematics curriculum of the MoNE. It broadcasts on three different channels: primary, middle, and high school, and each grade level has its own lesson schedule in each channel. The mathematics lecture videos for EBA-TV were created by experienced mathematics teachers. Students can access recorded lessons to review multiple times if needed. Although television creates access to mathematical learning, there are limitations. For instance, due to the diverse social, economic, and academic background of students in Turkey, it is difficult to create lessons that are responsive to students’ needs, cultural background, mathematical readiness, and achievement. Thus, we examined how mathematics teachers attend to equity (National Council of Teacher of Mathematics (NCTM), 2014) during the pandemic via synchronous modes of instruction and the lessons broadcast on television. We adhered to a theoretical standpoint for equity in mathematics education and acknowledge inequities that exist within the Turkish settings.

### Theoretical standpoint: equity in mathematics education

In Turkey, the dominant culture of mathematics teaching is mainly teacher-centered and prioritizes students who are successful at mathematics and labeled as intelligent and capable (Baki, 2020; Temizöz & Özgün-Koca, 2008). The majority of the research studies (Kabael, 2019; Yetkiner Özel et al., 2013) and policy reports (Karip, 2020; Ölçme, Seçme ve Yerleştirme Merkezi (The Measurement, Selection and Placement Center), 2020; TEDMEM, 2020) in Turkey focus on comparing high-stakes test results to illustrate inequities in mathematics achievements among student groups (e.g., gender and socioeconomic status). However, focusing solely on achievement gaps further amplifies negative stereotypes as to who can excel in mathematics and hinders the extent that all students are afforded equitable learning opportunities (Gutiérrez, 2008; Wilson, 2016). The disparities between student groups in high-stakes tests catalyzed reform movements in curriculum and policies in Turkey (MoNE, 2013, 2018a, b; Öztürk & Özmantar, 2019).

Although there are references to equitable mathematics teaching (e.g., affording equitable opportunities to students and high expectations for all students) in reform curricula, researchers found that mathematics teachers’ practices are resistant to change, and teachers struggle to enact the reformed curriculum as intended (Chapman & Heater, 2010). Moreover, only a few studies (Allexsaht-Snider et al., 2020), conducted in Turkey, focused on equitable mathematics teaching. Thus, we used the National Council of Teachers of Mathematics’ (NCTM) (2014) access and equity position statement in mathematics which entails the aspects emphasized in the Turkish mathematics curricula that attends to equity (Öztürk & Özmantar, 2019; Umay et al., 2006). We also used the NCTM’s (2014) access and equity position statement due to the contextual similarities between the USA and Turkey and deemed it appropriate for the Turkish context. Particularly, in both countries, teachers have second language learners in their mathematics classrooms (Payán & Nettles, 2008; Göç İdaresi Genel Müdürlüğü (Directorate General of Migration Management), 2016), ethnic diversity (KONDA, 2011; National Center for Education Statistics, 2020), and disparities in equitable access to education for students from different socioeconomic status (Gates, 2020; Yetkiner Özel et al., 2013).

The NCTM (2014) suggested that teachers should facilitate equitable opportunities in mathematics teaching and learning. According to the NCTM (2014) access and equity position statement, to create, sustain, and support equitable mathematics instruction requires

Being responsive to students’ backgrounds, experiences, cultural perspectives, traditions, and knowledge when designing and implementing a mathematics program and assessing its effectiveness… Addressing equity and access includes both ensuring that all students attain mathematics proficiency and increasing the numbers of students from all racial, ethnic, linguistic, gender, and socioeconomic groups who attain the highest levels of mathematics achievement. (p. 1)

It is recommended that teachers attend to aspects of equity within their practices. Culturally responsive teaching could be used to support equitable opportunities in mathematics for culturally and linguistically diverse learners (Abdulrahim & Orosco, 2020; Gay, 2018). Gay (2018) defined culturally responsive teaching as “using the cultural knowledge, prior experiences, frames of reference, and performance styles of ethnically diverse students to make learning encounters more relevant to and effective for them. It teaches to and through the strengths of these students” (p. 36). Culturally responsive mathematics teachers are effective in leveraging students’ mathematical understanding by creating a learning environment that allows them to use their primary language and providing curriculum materials responsive to their diverse needs (Abdulrahim & Orosco, 2020; Aguirre & Zavala, 2013; Gutiérrez, 2002).

Although schools are challenged to attend to equity in their design, practices employed, and how they allocate resources (Field et al., 2007), government and communities should seek to provide students access to resources that can ensure students advance in mathematics (Gervasoni & Lindenskov, 2010). Nevertheless, the extent resources are utilized may be due to teachers’ beliefs about how to facilitate instruction (Philipp, 2007). Hence, teachers’ beliefs (which may be productive or unproductive) and expectations play critical roles in how students are afforded access to high-quality mathematics instruction (NCTM, 2014). Ellis (2008) stated that teachers expect some students to engage in “a variety of mathematics topics through multiple teaching and learning strategies” by setting high expectations (NCTM, 2014, p. 61). Yet, teachers with unproductive beliefs may utilize labels to limit some students, particularly those in “low track,” from learning rigorous mathematics (Ellis, 2008). Thus, it is important to maintain high expectations for all students and make curricular decisions that promote equitable learning opportunities. For instance, teachers could adjust their instruction to empower students’ mathematical learning by considering students’ mathematical readiness, experiences, and needs (Cal & Thompson, 2014; NCTM, 2014) and carefully select a curriculum and pacing guide (Christenson & Wager, 2012; NCTM, 2014).

Moreover, as a result of COVID-19, the shift to remote instruction amplified barriers that impacted how students learn mathematics (Adams, 2020). The digital divide also privileges some students, while oppressing others, who may not have access to resources (Hohlfeld et al., 2017). Therefore, in examining how teachers attend to equity, consideration was given to factors that may influence their practices.

### Inequities that exist in Turkish mathematics context

In Turkey, inequities exist in mathematics such that students’ success is often correlated to their gender, ethnicity, linguistic background, and socioeconomic status (Baysu & Ağırdağ, 2019; MoNE, 2015; Tunga et al., 2020; Yetkiner Özel et al., 2013). Various studies (Çiftçi & Çağlar, 2014; OECD, 2019; Yetkiner Özel et al., 2013) have documented that students’ mathematical achievements are influenced greatly by the socioeconomic status of families. The underlying reason for this outcome is that families with higher socioeconomic status can provide their children with more opportunities (e.g., higher quality schools, educational materials, private tutoring, access to teachers, network) that contributes to success in mathematics and their future endeavors (Çiftçi & Çağlar, 2014; Yetkiner Özel et al., 2013). Additionally, according to a MoNE (2015) report, there is an achievement gap between eastern and western regions of Turkey in terms of mathematics scores, which can be explained by the income gap between the regions (eastern, low; western, high) (Çiftçi & Çağlar, 2014). Thus, in the Turkish setting socioeconomic status contributes to inequities in students’ mathematics learning opportunities and achievement.

Inequities also exist relative to cultural and language differences. In Turkey, there are various ethnicities, such as Turkish (70–75%), Kurdish, and Zazaki (18%, about 13 million to 18 million) (Aydın & Özfidan, 2014). Kırdar (2009) reported that Kurdish children are often economically disadvantaged, and Kurdish children between the age of 8 and 15 years old are about twice as likely to not enroll in school compared to Turkish children. Also, the Syrian refugee population experiences educational inequities (Aydın & Kaya, 2017; Tunga et al., 2020). According to UNICEF (2017), nearly 40% of Syrian children (380,000) are out of school. Moreover, over 64% of the urban Syrian households live close to or below the poverty line, with school age children working in low-wage jobs instead of attending school. Of the Syrian and Kurdish children that are in school, there are multiple barriers that impede their learning such as cross-cultural clashes, academic performance below grade level, and language barriers (Kaya, 2015; Nielsen & Grey, 2013).

Inequities are also compounded by teachers’ values and cultural differences that may exist between teachers and their students, which may limit the extent teachers intentionally disrupt inequities within mathematics (Dede, 2015). Therefore, considering the inequities that exist and efforts to provide equitable learning opportunities for mathematics via the Internet and/or television for students in Turkey, there is a need to examine how these mediums are used to facilitate mathematics teaching and learning during the pandemic for all students, particularly disadvantaged students. In this study, disadvantaged students are the ones with limited learning opportunities in schools due to their families’ socioeconomic or social status, mathematical readiness and success, and language barriers. Thus, this study aims to examine mathematics teachers’ perspectives on factors that support or hinder how equity is attended to in mathematics during remote instruction compared to face-to-face instruction, particularly for disadvantaged students. This paper answers the following research questions:

1. What are teachers’ perspectives on factors that support or hinder equitable mathematics teaching and learning opportunities for disadvantaged students during remote instruction delivered through EBA-TV and synchronous lessons with consideration of how these practices are similar or different from face-to-face instruction?
2. How do teachers attend to equity in mathematics for students who are non-native Turkish speakers via face-to-face and remote instruction?

In both research questions, we seek to unpack factors that can impact the extent equity is attended to in remote mathematics instruction from the theoretical standpoint discussed previously.

## Method

Interviews were used to gain an in-depth understanding (McNamara, 2009) of mathematics teachers’ perspectives on factors that hinder or support equity in mathematics instruction via remote and face-to-face modes of instruction. “We use the term perspective to postulate a broad pedagogical structure composed of multiple conceptions that collectively organize some aspects of a teacher’s practice” (Tzur et al., 2001, p. 228). Thus, by capturing teachers’ perspectives we can unpack the underpinnings of their instructional decisions and practices to address how they attend to equity in mathematics. An interview allows researchers to see the “centrality of human interaction for knowledge production, and emphasizes the social situatedness of research data” (Kvale, 1996, p. 4). Therefore, the use of interviews provided insights into the teachers’ perspectives.

### Sample

Convenient, yet purposeful, sampling was used to select nine mathematics teachers to be interviewed. The participants were selected from readily available but information-rich cases related to the phenomenon of interest (Creswell, 2013). Teachers were invited to participate if they worked in different educational settings in Turkey (e.g., different grade levels, school location, multi-graded or mono-graded classroom) and if they taught mathematics to students with different ethnicities, socioeconomic status, and language barriers. This selection would provide an in-depth understanding on how teachers from different settings perceive equity. Table 1 shows the information provided by the teachers regarding their respective settings relative to students’ socioeconomic status, ethnicity, language barriers, and access rates.

**Table 1:** Demographic information regarding the teachers and their respective settings

Teacher	Grade level taught	Experience (in years)	School location	Class type	Students’ (STs) ethnicity	STs’ socioeconomic background	STs’ with language barriers	EBA-TV access rate (%)	Synchronous lessons-access rate (%)
T1	2nd and 4th	12	Rural	Multi-graded	Turkish	Low	No	100	0
T2	1st and 2nd	15	Rural	Multi-graded	Turkish Kurdish	Low	No	8	0
T3	3th	3	Rural	Mono-graded	Turkish Zazaki	Middle	Yes	100	80^a^
T4	1th	23	Urban	Mono-graded	Turkish	High	No	100	100
T5	6th and 7th	3	Urban	Mono-graded	Turkish Syrian	Low	Yes	50	0
T6	8th	5	Rural	Mono-graded	Turkish	Middle	No	95	60
T7	5th and 8th	5	Urban	Mono-graded	Turkish Syrian	High	Yes	100	100
T8	9th and 11th	4	Rural	Mono-graded	Turkish	Low	No	Not provided	0
T9	High school	27	Urban	Mono-graded	Turkish	Middle	No	100	70

^a^The teacher increased the access rate to 100% by addressing the problem

### Data collection

Three researchers created the interview protocol based on essential aspects of equitable mathematics instruction (e.g., setting high expectations of all students, culturally responsive teaching) that was relevant to the Turkish context and aligned with the NCTM’s (2014) access and equity position statement. After we finalized the protocol, the fourth researcher provided feedback on the protocol. The initial protocol was piloted with an elementary school teacher, and the data garnered were analyzed by the researchers to see which questions provided in-depth responses. After the analysis, the researchers decided to combine some of the questions to enhance the quality of the interview. This adjustment also ensured the duration of the interview did not exceed an hour. Table 2 shows example questions for the interview protocol sections.

**Table 2:** Example of interview questions

Sections	Face-to-face instruction	Remote instruction
Curriculum and instruction	What kind of teaching strategies do you utilize to ensure equity and accessibility in face-to-face mathematics instruction? How do you integrate the student’s culture to the math class?	To what extent do you think you manage to implement the teaching strategies you have just mentioned in the distance/remote education environment? How do you integrate the student’s culture to the mathematics class? To what extent is the remote instruction environment offered by the Ministry of National Education sensitive to the local culture and needs of students?
High expectation for all students	What do you expect to see in a classroom where high-quality mathematics instruction is carried out? (elaborate on teachers’ expectations on students’ mathematical capabilities, nature of task, student engagement, etc.)	To what extent do your students have an opportunity to access high-quality mathematics instruction in the remote mathematics teaching environment offered to students due to COVID-19? Why? (elaborate on teachers’ expectations on students’ mathematical capabilities, nature of task, student engagement)
Support mechanism and access to resources	What kind of support mechanism do you utilize to ensure equity and accessibility in face-to-face mathematics instruction? (NCTM, 2014) What do you do to ensure equitable and accessible mathematics learning opportunities for students: • with a language barrier? • with low socioeconomic status? • with mathematical learning difficulties? • with different mathematical achievement levels?	What kind of support mechanism do you utilize to ensure equity and accessibility in remote mathematics instruction? (NCTM, 2014) What mechanisms are provided for students with language barriers to ensure access to equitable mathematics learning in remote instruction? What resources are provided for students with low socioeconomic status to ensure access to equitable mathematics learning in remote instruction? What do you do to ensure equitable and accessible mathematics learning opportunities for students: • with a language barrier? • with low socioeconomic status? • with mathematical learning difficulties? • with different mathematical achievement levels?

It is our stance that high-quality instruction should be equitable such that all students feel included and are afforded access to engage in rigorous mathematical activities (Munter, 2014). We are aware that the concept of equity is bounded with individual interpretation and cultural and political context (Bartell et al., 2017; Gates, 2020), and it can be taken up and operationalized in multiple ways. Thus, to address this complexity, we shared the NCTM’s (2014) access and equity position statement in the consent form with the teachers. Also, we asked teachers what they do to ensure equitable mathematics instruction in face-to-face and online settings and what high-quality mathematics instruction means as initial questions for the interview. These questions gave us an understanding of each teacher’s perspective of equity and high-quality mathematics teaching.

Three researchers conducted the interviews with teachers over the phone or Zoom. The teachers who participated in the study submitted informed consent via Google forms.

### Data analysis

Data analysis was organized in four phases. In the first phase, the interviews were transcribed, and we examined the transcribed data to identify the text segments (DiCicco-Bloom & Crabtree, 2006) in which teachers talked about equity in mathematics.

In the second phase, we conducted an initial inductive analysis of text segments to identify how teachers shared their perspectives on equity in mathematics. We also grouped teachers’ responses into larger categories of curriculum and instruction, support mechanism, and access to resources based on relevant literature (Cal & Thompson, 2014; Gay, 2018; Gutiérrez, 2012; NCTM, 2014). For instance, we identified curriculum and instruction as the main category to capture teachers’ perspectives relative to quality of mathematics instruction (Gutiérrez, 2012; NCTM, 2014) and curricular decisions (Cal & Thompson, 2014).

In the third phase, we engaged in an open coding process to determine sub-categories and related factors that support or hinder equity in mathematics instruction (Gibbs, 2018). This analysis yielded multiple factors such as student engagement (interaction), pacing, depth, and language. Subsequently, we worked on refining and merging the related factors. For instance, student engagement (interaction), attaining students’ thinking and understanding, nature of the task, and teacher factors were categorized under teachers’ vision of high-quality mathematics instruction. For this instance, Munter’s (2014) study on high-quality mathematics instruction informed our refining and merging decisions. The refinement of other factors and sub-categories was also informed by existing literature on equity in mathematics (see Table 3).

**Table 3:** Main categories, sub-categories, and factors

Main Category	Description	Sub-category	Factors
Curriculum and instruction	Teachers’ perspectives on the ways to create access to “all students to a high-quality mathematics curriculum, effective teaching and learning” (NCTM, 2014, p. 1); responsiveness to students’ culture, needs, diverse backgrounds, mathematical knowledge (Gay, 2018) and “teachers’ involvement in curricular decisions” (Cal & Thompson, 2014) Ensuring high-quality mathematics instruction for all students requires high expectations for all students (Gutiérrez, 2012). Teachers’ belief of “All students are capable of participating and achieving in mathematics, and all deserve support to achieve at the highest levels” (NCTM, 2014, p. 63)	High-quality mathematics instruction (Gutiérrez, 2012; NCTM, 2014)	Teachers’ vision of high-quality mathematics instruction (Munter, 2014) Student engagement (interaction) Attaining students’ thinking and understanding Nature of the task Teacher factor Teachers’ high expectations for all students (Gutiérrez, 2012; NCTM, 2014)
		Culturally responsive mathematics teaching (Gay, 2018)	Cultural experience (Aguirre & Zavala, 2013; Civil, 2006) Language (Aguirre & Zavala, 2013; Civil, 2006)
		Teachers’ involvement in curricular decisions (Cal & Thompson, 2014)	Adjusting curriculum and instruction according to students’ experience, mathematical readiness, and needs (Christenson & Wager, 2012; NCTM, 2014) Pacing Depth
Support mechanism	Teacher perspectives on the ways of giving the differentiated support and opportunities for all students regardless of their ethnicity, learning barriers, and socioeconomic status to ensure their equal access to mathematical learning (NCTM, 2014)	N/A	After school activities (NCTM, 2014)
			Extracurricular activities (NCTM, 2014)
			Allocating extra time (Burris et al., 2008).
			Peer learning (O'Donnell & King, 2014)
Access to resources	The ways of ensuring all students have the access to the same curricular materials (Cal & Thompson, 2014; Gutiérrez, 2012; NCTM, 2014)	N/A	Access to technology (computer etc.) (Cal & Thompson, 2014)
			Access to manipulatives (Cal & Thompson, 2014)
			Access to Internet
			Access to teacher who teaches mathematics
			Access to same amount of lesson time (Cal & Thompson, 2014)

In the fourth phase, researchers independently coded text segments into the saturated categories and factors. In some instances, one text segment was coded in multiple categories. These codings not only represent patterns in teachers’ perspectives on equity but also reflect the essential aspects of equity in the existing mathematics education literature (see Table 3). Then, the researchers discussed their codings, and the consistency rate was found to be at 87% (Miles & Huberman, 1994). Revisions were made in the coding until the four researchers came to an agreement. Member checking was used to clarify the meaning of the teachers’ responses when researchers had different interpretations of the response.

## Findings

The findings suggest that all teachers perceived remote instruction that broadened existing inequities in mathematics due to lack of access to resources, limited time for students to communicate mathematically, regional constraints, and challenges to enact culturally responsive teaching in mathematics. The teachers also acknowledged that there are some benefits of remote instruction, particularly related to EBA-TV. Factors that impacted the extent teachers attend to equity included access to resources; support mechanisms inclusive of socioeconomic status, mathematical readiness of students, and students with language and cultural barriers; and teachers’ expectations and curricular involvement.

To structure the findings, we will describe the teachers’ perspectives on the factors that support and hinder equity and accessibility in mathematics teaching, in remote and face-to-face modes of instruction. We will also describe strategies used to attend to equity, particularly for students with language barriers.

### Factor hindering and supporting equity: EBA-TV

We describe teachers’ perspectives on the factors that hinders and support equitable mathematics instruction when EBA-TV is used under two main categories: access to resources and curriculum and instruction.

#### Access to resources

Eight out of 9 teachers believed that mathematics instruction was conveyed equitably with some limitations through EBA-TV. Particularly, the majority of teachers (except T4) suggested that if their students watched EBA-TV, it would reinforce the content taught face-to-face. However, T2 highlighted that the majority of her students (92%) living in a rural village still do not have access to television, which made it impossible to access mathematics lessons. Also, two teachers stated that in very crowded families (i.e., 20 people in one home), the students do not have a chance to access the television for learning mathematics even if the household had one television. For example, a teacher stated:

T2: When I spoke with one of my students on whether s/he watched [mathematics lesson in EBA-TV]. My student responded “I could not watch the channel I wanted. Even if I watched since the home is quite crowded, I could not understand the topics”.

T2: Öğrencimle konuştum EBA-TV’yi izliyor musun diye. Öğrencim “İstediğim kanalı izleyemiyorum. İzlediğimde ise ev kalabalık olduğu için, konuları anlamıyorum.” dedi.

As T2 indicated, not all students are able to take advantage of EBA-TV, especially those from low-income families. These students lose their opportunity to learn mathematics during remote instruction.

Four teachers (T1, T2, T3, T6) stated that access to technology and manipulatives posed a challenge in facilitating equitable opportunities in face-to-face instruction. T1, T3, and T6 mentioned that it was difficult to attend to visualization in mathematics without the smart boards used in EBA-TV. For instance, T1 stated advantage of accessing remote instruction as:

T1: The visual of the 3D objects, which I used to have difficulty with showing and explaining, presented very nicely with the smart board in there [EBA-TV].

T1: Akıllı tahtadan 3 boyutlu bir cismin görselini ben burada anlatamazken o orada [EBA-TV] çok güzel şekilde öğrencilerime gösterebiliyor ve anlatabiliyor.

Two teachers (T1, T5) also acknowledged that access to the same quality mathematics teacher is a factor for equity. This opportunity is especially important for the students who do not have access to a teacher who teaches mathematics in their schools during face-to-face instruction. For instance, T1 just started to teach mathematics to second graders, and prior to his teaching, these students did not have much opportunity to learn mathematics in school. Thus, learning mathematics from a mathematics teacher is a privilege. Another teacher (T5) commented on the quality of the mathematics instruction in relation to teachers’ effectiveness as:

T5: In face-to-face instruction there are teachers teaching mathematics worse or better than me, this creates inequality. But now all students learn mathematics from the same teacher with the same instructional content*.*

T5: Benden daha iyi ya da daha kötü anlatanlar vardır yüz yüze eğitimde, bu dezavantaj durumu yaratıyordu. Ama şu an herkes matematiği aynı öğretmenden, aynı konuyu, aynı şekilde öğreniyor.

Thus, access to technological resources and effective teachers were identified as contributing factors to equitable learning opportunities.

#### Curriculum and instruction

All teachers (*n* = 9) acknowledged the implications of pacing in attending to equity. For instance, the start of the mathematics topic on EBA-TV did not align with their current place in the horizontal curriculum trajectory. Five teachers (T1, T2, T5, T6, T8) who were working in rural schools and have financially and academically struggling students indicate that their students’ mathematical readiness is not being met by EBA-TV lessons. T2 noted:

T2: We were far behind in the curriculum. So, they [my students] don’t understand. We couldn’t even finish all four operations with second graders. Lessons [in EBA-TV] are more beneficial to children who study in urban schools. We repeat topics three or four times for students in the multi-graded instruction classes.... So it’s not pacing [in EBA-TV lessons] where we can catch them.

T2: Müfredatta biz baya gerideydik. O yüzden anlamıyorlar. Dört işlemi bile ikinci sınıflarda bitirememiştik. Ders anlatımı, işlenişi EBA TV'de daha çok merkezde okuyan çocuklara daha faydalı. Birleştirilmiş sınıftaki öğrenciler için konuları üç dört defa tekrar ediyoruz….O yüzden onları yetişebileceğimiz bir hız değil.

As seen in the comment, T2 is far behind the topics instructed in EBA-TV. The responses of these teachers also showed that (1) teaching in a multi-graded instruction class resulted in half of the mathematics lesson time being allocated to mathematics, compared to mono-graded instruction class, and (2) having limited access to mathematics teachers and/or (3) not prioritizing students’ mathematics learning due to economic hardship of the family led to this difference in readiness level. These teachers indicated that EBA-TV lacks structures to give individualized mathematics learning opportunities to close knowledge gaps that exist.

Three teachers (T1, T2, T8) who teach mathematics to students with low socioeconomic status in disadvantaged schools raised concerns about the depth of mathematics being addressed, due to limited conceptual explanations of the topics and procedural steps being skipped in EBA-TV. The teachers stated that this situation creates inequities mainly for the students who had learning difficulties or low mathematical achievements. Also, the teachers acknowledged that limited opportunities were provided to review a concept and that there were few tasks posed, which hindered equitable learning opportunities. For instance:

T8: The topics in [EBA-TV] are explained more superficially, meaning steps could be missed.... In face-to-face class, we explored every step with my students who had low achievement. This ensures active implementation of mathematics.... The majority of my students had prior knowledge gaps. Now, in EBA-TV when the steps were skipped I am sure 90% of my students asked, “Where does this come from?”

T8: Şöyle, [EBA-TV] biraz daha yüzeysel geçiyor, yani ara işlemler atlanabiliyor.... Yani bir sınıfta en küçük ara işlemi bile açıklıyoruz belki benim öğrencilerimin seviyesi düşük olduğu için. Böyle aktif bir matematik götürebiliyorum.... Çocuklarımın çoğunun temelden gelen bir eksikliği var. Şimdi EBA-TV’de ana işlemler atlandığı zaman %90 benim öğrencilerim için, “Ya bu nereden geldi ki buraya?” cümlesini kullandıklarından eminim yani.

These teachers (T1, T2, T8) emphasized that the mathematics conveyed on EBA-TV may be difficult for students to understand and highlighted knowledge gaps in mathematics.

The gap in opportunities for mathematics learning between students from high socioeconomic status in urban schools became more evident during remote instruction when considering the mathematics lesson pacing, depth, and its appropriateness for students’ mathematical readiness. Four teachers (T3, T4, T7, T9), who worked in urban and economically advantaged schools, stated that the mathematics lesson content in EBA-TV is suitable for their students’ mathematical readiness, in some cases it was even below their level. These teachers also gave synchronous lessons to make mathematics instruction more rigorous considering EBA-TV lessons were below their students’ mathematical readiness. For instance:

T4: My kids are good. The statements from my students who have math intelligence and mathematics competencies are; what is this? It is child play [too easily]. We have already done this.... One of my students asked me “Do you see value in watching this?”

T4: İyi çocuklarım. Bunlar sayısal zekası önde giden ve becerebilen çocuklarımın söylediği laflar aynen bu; Bu ne ya diyorlarmış, çocuk oyuncağı, biz bunları zaten yapıyoruz.... Öğrencilerimden bir tanesi “Siz izlemeye değer görüyor musunuz?” diye sordu direk bana.

This quote also showed that the content of EBA-TV lessons is perceived as minimally meeting the needs of students with more privileged academic backgrounds. Teachers indicated that their students who have high mathematics achievement and are from high socioeconomic status do not see the value in watching the mathematics lesson in EBA-TV, which significantly contrasts the perspectives of the level of rigor of the content for students with low mathematics achievement and low socioeconomic status. Thus, one can say that there is no space for adjusting curriculum and instruction according to students’ mathematical readiness and needs in EBA-TV. Also, the socioeconomic status of the families greatly influenced a teacher’s involvement in curricular decisions to design high-quality mathematics instruction that is accessible to all students.

The majority of the teachers (*n* = 6, T1, T2, T3, T4, T5, T8) acknowledged that EBA-TV does not readily exhibit culturally responsive mathematics teaching. These teachers indicated that mathematics lessons in EBA-TV do not integrate students’ local culture, language, and experience which resulted in low-quality mathematics teaching, when compared to face-to-face instruction. The teachers believed that integrating cultural context in mathematics supported students’ mathematics learning in particular for the concepts they had difficulty in understanding. For instance:

T1: There is harvest in the village where my school is located.… They had great interest in combine harvesters and knew a great deal about it. [In face-to-face] they had difficulty in calculating the perimeter and area of rectangles or polygons when we explained procedurally. Instead we did this: In the school-garden one kid became a harvester, the kid became a driver. They draw a rectangular farm. I said to them “You will harvest this farm but first let’s walk around the garden, then calculate the perimeter and the area of the garden. The kid held his peers’ hand and thought as a harvester and walked around the farm…. Then, in the classroom we reflected back to this experience when working on the area and perimeter of polygon problems in class. They solve these problems.

T1: Okulumun bulunduğu köyde biçerdöverlik var.... Biçerdövere müthiş bir ilgileri var ve biçerdöverle ilgili her şeyi biliyorlar. Şimdi [yüz yüze eğitimde] dikdörtgenin ya da çokgensel şeylerde çevreyi ve alanı saplarken hesaplamak olarak anlattığınızda çocuk anlamıyor. Biz şunu yaptık: Bahçeye çıktık bir arkadaşı biçerdöver oldu, diğer arkadaşı şoför oldu. Bahçeye büyük bir dikdörtgen tarla çizdik. Ben dedim ki; “bu tarlayı biçerdöver ile sen işleyeceksin ama önce bunun çevresini bir etrafını dolanalım bakalım ne kadar bir çevre ve alan bunu hesaplayalım”. Çocuk arkadaşının ellerinden tuttu ve onu biçerdöver olarak düşündü ve çizdiğimiz tarlanın çevresini dolaştı geldi…. Daha sonra sınıfta çokgenlerin alan ve çevre problemlerinde bu deneyime baktık. Bu problemleri çözebildiler.

T1 adjusted his mathematics instruction to be responsive to students’ cultural experience. As a result, students can understand the topics that they had difficulty with. This teacher also stated his expectations for all students as “when you give the appropriate opportunities, they could do mathematics.” Due to no access to the Internet, he could not adjust the remote instruction to create learning opportunities responsive to students’ needs, academic achievement, and cultural experiences as he did face-to-face.

### Factor hindering and supporting equity: synchronous lessons

This section reports on the teachers’ perspectives on factors that hinder and support equitable mathematics instruction delivered with synchronous lessons under two main categories: access to resources and curriculum instruction.

#### Access to resources

Five (T3, T4, T6, T7, T9) teachers acknowledged that their students had access to synchronous lessons. However, synchronous lesson access rate varied. Only T4 and T7’s students had 100% access. This showed that access to the Internet and technology played out differently for T3, T6, and T9. The following quotes highlight teachers’ difficulty in addressing challenges for ensuring access for all students.

T9: Many parents called and said...we have no financial power to connect to the Internet, so we cannot attend classes.... We are inadequate to [solve] the issue. If the students’ financial levels are the same, then we may have provided equal opportunities. ...Even if it is remote, we could have done good studies.

T9: Benim birçok velim aradı…evimizde internet yok bağlatacak maddi gücümüz yok, o nedenle biz derslere katılamıyoruz diye mesajlar attılar…. Biz o konuda [çözüm üretmekte] yetersiz kaldık. Şimdi öğrencilerin maddi düzeyleri aynı olsa o zaman fırsat eşitliğini sağlamış olabiliriz. ... Eğitim uzaktan da olsa güzel bir çalışma yapılabilirdi.

T3: ...I have a very successful student without access to the Internet.... I found my own methods.... I solved the access problem of this student by talking to the principal.

T3: …İnterneti olmayan akademik olarak başarılı bir öğrencim var.... Ben kendi yöntemlerimi buldum…. İnternete ulaşımı olmayan bir öğrencimin erişim sıkıntısını müdür ile konuşarak çözdüm.

Both teachers stated that there were inequities within their own classrooms as a result of the socioeconomic status of the students. When few students’ learning is impacted because of financial constraints as in T3’s case, the teacher can address the access problem more easily. However, when the inequity is wide, as in T9’s case, it becomes very hard for an individual teacher to ensure all students have access to mathematics during remote instruction.

After T3 addressed the access problem for her student, her access rate was raised to 100%. Thus, T3, T4, and T7 have access to resources, such as the Internet and technological tools, to do synchronous lessons at the same rate. However, the way that they use these resources differed due to their curricular decisions, vision of high-quality mathematics instruction, and expectations from students.

#### Curriculum and instruction

The teachers (T1, T2, T5, T8) whose students only had access to EBA-TV hypothetically added that if their students had access to the Internet and technology, they would have more involvement in making curricular decisions that are responsive to their students’ needs, mathematical readiness, and cultural experiences. Thus, they could incorporate culturally responsive teaching practices and interactive and real-world tasks in which their students interact with each other. These teachers believed that their students do not have equitable opportunities to learn mathematics during remote instruction compared to students who had access to the Internet and synchronous lessons. For instance:

T5: Only two students asked me ‘Can we do it? [Live lesson]’. Anyway, this is a girl whose older brother is a doctor... but I haven’t done anything. Other children said ‘We do not have. What will we do?’. They get into something [feeling] like this. They heard from each other. [If I give lessons to these two], I have destroyed the equal opportunity [for all]. In fact, I should give [lesson] to these students.

T5: Şu ana kadar iki kişiden “Hocam biz [canlı ders] yapabilir miyiz?” talebini aldım. Zaten bu dediğim bir abisi doktor olan kız çocuğu... ama daha şey yapmadım. Diğer çocuklar “Bizim yok. Biz ne yapacağız?”. Böyle bir şeyin [duygunun] içerisine giriyorlar. Birbirlerinden duyuyorlar. [Eğer bu ikisine ders verirsem], [herkes için] fırsat eşitliğini ben çökertmiş oluyorum. Bilmiyorum aslında [ders] yapmalıyım sanırım o öğrencilere.

The quote shows how students thought and felt about this inequity. Also, it showed the underlying reason for this inequity as the socioeconomic status of the student. Due to the different socioeconomic statuses among T5’s students, she struggles in making curricular decisions. The teacher dealt with the dilemma of facilitating live instruction for a few of her students, knowing other members of the class are unable to participate.

Five teachers (T3, T4, T6, T7, T9) who can do synchronous lessons stated that this is a great chance for creating equitable mathematics learning opportunities for their students. Nevertheless, they acknowledged that student engagement cannot be fully realized in the same quality in synchronous lessons compared to face-to-face. Lack of hands-on tasks implemented (*n*=2), and the teachers could not circulate the class, so it is hard for teachers to fully attain students’ mathematical thinking and understanding during synchronous lessons (*n*=5). All teachers stated that when they circulated the classroom, they could hear students’ thoughts and observe non-verbal cues as to whether a student understands. However, the remote instruction reduces their ability to capture students’ mathematical thinking and understanding. The teacher’s vision of high-quality mathematics impacts how they respond to the constraints of remote instruction. For instance, teachers noted:

T7: Before distance education, I used to bring, for example, an angle gauge, a protractor, ...to the class. I was giving it [the tools] to the student’s hand, ...which s/he can learn by hands on experience... But, I can’t do this during remote education. I can only show it on the interactive board or applications. The students do not have a chance to perform.

T7: Uzaktan eğitim öncesinde derse mesela bir açı ölçer, bir gönye olsun, bir iletki olsun, ...sınıfa getiriyordum. Öğrencinin eline veriyordum, ...hani kendisi onu yaparak yaşayarak öğrenebiliyor… Ama şu anda uzaktan eğitim sürecinde bunu yapamıyorum. Sadece etkileşimli tahta ya da uygulamalar üzerinden gösterebiliyorum şu anda bunu. Öğrencilerin pek yapma imkanı olmuyor.

T3: When I talk about line, plane, line segment, a board appears on the screen. ...We don’t have a smart board in our elementary schools. But now we can use the smart board feature you know during the live sessions.... So, while I am performing a live session, ...I make a few interactive examples. So, they are completing what I share, or a friend can complete a work done by another peer. In this sense, I see a positive [influence]… especially in the math class.… In the sense that the children can engage and interact with, this increases the quality of mathematics.

T3: Mesela matematik dersinde ben işte doğru, düzlem, doğru parçası bu konuları anlattığım zaman ekranda bir tahta çıkıyor. Tabi, mesela şöyle sınıfta akıllı tahtamız yok bizim ilkokullarda. Ama şu an canlı ders esnasında bildiğin akıllı tahta özelliğini kullanabiliyoruz.... Yani canlı ders yaparken, ...etkileşimli birkaç tane örnek yapıyorum. Yani benim paylaştığım şeyi onlar tamamlıyorlar ya da bir arkadaşının yaptığı bir çalışmayı arkadaşı tamamlayabiliyor. Bu anlamda bir artısını gördüm… özellikle matematik dersinde. Çocukların birebir kendilerinin içine girdiği kendilerinin etkileşim içerisine girebildiği, bu anlamda matematik niteliğini artırdığını düşünüyorum.

T7’s response showed that she had difficulty in engaging her students in synchronous lessons since she could not bring hands-on materials to these lessons as she did in face-to-face instruction. Although T3 thought there are challenges (not bringing concrete materials, attending students’ mathematical thoughts) in students’ engagement in synchronous lessons, this teacher took advantage of existing features provided within remote instruction to which she did not have access in face-to-face instruction. Another striking example for this was how T4 made curricular decisions that resulted in lowering his expectations for the students in remote mathematics instruction. T4 indicated that he implemented challenging tasks, hands-on activities, etc. to teach mathematics in face-to-face instruction. Yet, he did not do any of these in remote instruction since he believed his students’ mathematical competencies were high enough to progress to the next grade level. Thus, he only solved multiple choice mathematics problems with each student 10 min per week.

#### Support mechanism: EBA-TV and synchronous lesson

All teachers used at least one type of support mechanism in face-to-face mathematics instruction. However, they could not use any of these support mechanisms (e.g., peer learning) for their struggling students in remote instruction through EBA-TV because EBA-TV did not allow these teachers’ involvement in curricular decisions and student interaction. Also, none of the teachers who did synchronous lessons stated that they used any support mechanism. Only T1 whose students did not have access to the Internet stated that if he had access to the Internet “He would assign after-school mathematical activities.”

Teachers’ responses showed losing access to support mechanisms in remote instruction resulted in inequitable learning opportunities for students compared to face-to-face instruction. One example is related to the commonly used (*n*=5) approach to peer learning:

T5: I chose 10 students from high, medium achievers. Then, I had them sit with a more unsuccessful student, then [told] ‘Let’s teach him/her’. We were doing it at the end of the units, even if it was a couple of hours a week. Now, this is not happening.... This was the loss of the middle and lower levels. Others [high achievers in math] did not lose anything, but they might like to do such a thing.

T5: Diyelim ben seçiyordum 10 tane iyi, orta seviyede öğrenciler. Onları ikili ikili yan yana oturup birinin yanına biraz daha başarısızı koyarak, “Hadi sen ona öğret” [diyorum]. Böyle bir şey yapıyorduk haftanın bir iki saat de olsa ünitelerin sonunda. Şu an mesela bu yok.... Bu tabii orta ve alt seviyenin kaybı. Diğerleri [başarılılar] bir şey kaybetmiyordu belki ama seviyorlardı böyle şey yapmayı.

T5’s students only have access to remote instruction via EBA-TV, thus they lost the peer learning opportunity that they had in face-to-face instruction.

All teachers used after school and extracurricular mathematics activities in face-to-face instruction for students struggling with understanding mathematics. For example, T2 stated that she organized “after school mathematics courses for the students who struggle in mathematics” and two teachers (T1, T3) organized for high achievers. However, none of them could do these activities in remote instruction.

The last support mechanism of allocating extra time for mathematics instruction was used by five teachers (T1, T3, T4, T5, T8) in face-to-face instruction. For example:

T4: We have time called a free activity lesson.... We treated some of these lessons as mathematics lessons and taught mathematics.

T4: Biz de serbest etkinlik dersi dediğimiz saatimiz var.… Bu ders saatlerinin bazılarını matematik işleyerek, matematik dersiymiş gibi işleyerek vermeye çalışıyoruz.

Overall, although teachers continued to provide instruction remotely as a result of the COVID-19 disruption of educational settings, the extent to which teachers operationalized support mechanisms varied based on limited access to the Internet or due to their personal decision making.

### Students with language barriers from diverse ethnicity: EBA-TV and synchronous lessons

All teachers agreed that the mediums in remote instruction do not ensure inclusion of students with language barriers in mathematics learning. Based on data analysis, students with language barriers are grouped under three categories: Zazaki (T3), Kurdish (T2), and Syrian (T5, T7) students. These teachers indicated that they used mainly peer learning as a support mechanism to overcome language barriers to ensure equitable mathematics instruction in face-to-face settings.

T3 works in a Zazaki village where Zazaish is spoken intensively. She stated that during face-to-face instruction, language barriers were not a huge challenge for her students because of group work and problem-solving activities. Although this is the case for third graders, she noted that first graders, who did not have enough time to learn Turkish, are struggling with understanding mathematics lessons delivered through EBA-TV. These first graders lack access to peer learning or extra-support activities during remote instruction, which they had had during face-to-face instruction.

T2 works in an eastern village where Kurdish is spoken extensively. She explained students’ difficulties as:

T2: A few of my students who can watch EBA-TV have problems. They don’t understand because they think in Kurdish. These problems also occur at school. Therefore, I am constantly trying to give different examples.... We don’t have many language problems in mathematics. We don’t have any problems because I use [concrete] materials. Now [in remote instruction] they don’t understand because they don’t have a chance for that. Since the family always speaks Kurdish at home, they can forget Turkish. We have these problems at the beginning of the year. I understand the children because I speak Kurdish.... Distance learning is not effective for students with a language barrier. When we explain the subject matter to these students, we get help from other students [who speak Kurdish].

T2: EBA-TV izleyen öğrencilerin bir iki tanesinde problem oluyor. Nedeni de Kürtçe düşündükleri için anlamıyorlar. Okulda da bu sorunlar oluyor. O yüzden sürekli farklı örnekler vermeye çalışıyorum.... Matematik konusunda çok dil sorunu yaşamıyoruz. [Somut] Materyal kullandığım için sorun yaşamıyoruz. Şimdi [uzaktan eğitimde] öyle bir şansları olmadığı için anlamıyorlar. Aile evde hep Kürtçe konuştuğu için Türkçe’yi unutabiliyorlar. Sene başında bu sorunları çok yaşıyoruz. Ben Kürtçe bildiğim için çocukları anlıyorum.... Dil bariyeri olan öğrenciler için uzaktan eğitim verimli olmuyor. Bu öğrencilere konu anlatırken diğer [Kürtçe konuşan] öğrencilerden yardım alıyoruz.

T2’s response illustrates that students experienced difficulties in understanding mathematics in remote instruction because they talked Kurdish, and the official instruction language is Turkish in EBA-TV. T2 addressed this barrier in face-to-face instruction by various support mechanisms such as peer learning, speaking Kurdish with them, and using concrete materials in mathematics.

Similar difficulties were stated by T5 and T7 who have Syrian students. T7 had one Syrian student in 8th grade. She explained her interaction with the students in face-to-face instruction as:

T7: ...He tries to sit very far back and at the corners [of the classroom]. I try to get him to sit next to students who are a bit more talkative to get him to join the class a little bit more. I checked...what he wrote. I try to explain as much as I can if he has difficulty in solving the problems.... I can only help this way ...I don’t think he understands much from what I say because he doesn't speak Turkish.

T7: ...[Sınıfta] Çok arkalarda ve köşelerde oturmaya çalışıyor. Onu biraz daha derse katılımını sağlamak için biraz daha konuşkan olan öğrencilerin yanına oturtmaya çalışıyorum. Defterini kontrol ediyorum yazdıklarını. Soru çözümünde zorlanırsa anlatabildiğim kadar anlatmaya çalışıyorum.... Ancak bu şekilde yardımcı olabiliyorum… Türkçe bilmediği için anlattıklarımdan pek fazla bir şey anladığını düşünmüyorum.

T7 stated that this student joined the live lessons but did not talk or engage in activities. She noted that the student “is quiet and shy” and has a language barrier. She did not provide any support or check the student’s mathematical understanding in remote instruction.

T3, who has students with language barriers and the same resources during remote instruction, teaches differently based on her expectations for her students and her beliefs about students’ mathematical capabilities.

T3: I have students who are successful in mathematics despite learning to read and write late. Language barrier is a problem but especially failure in mathematics is not related to this [language barriers]. As I said, I’ve experienced the opposite.

T3: Benim okuma yazmayı çok geç öğrendiği halde matematiği çok iyi olan öğrencilerim var. Dil problemi bir sorun evet ama özellikle matematik başarısı buna [dil bariyerine] bağlı değil. Dediğim gibi bunu aksi çok durum yaşadım.

Although T3 and T7 experienced the same challenge with language barriers, their reaction in face-to-face instruction to ensure high-quality mathematics instruction differed due to different expectations for their students. As seen in the responses, T3 set high expectations for all students and believed they can be successful in mathematics regardless of their language barrier. On the other hand, T7 attributes students’ difficulty in learning mathematics to students’ characteristics and language barrier. These teachers’ responses also showed that they held their expectations and beliefs in remote instruction. Thus, teachers’ belief about students’ mathematical capabilities and curricular decisions are critical to ensuring equitable mathematics teaching even if resources were not a factor.

T5 had several Syrian students and stated “The majority has language problems.” She offered after school activities and helped students with dual language, namely, Turkish and Arabic. Yet, her expectations for the students determined how she set the mathematical learning goal for the students:

T5: ...at least strengthen four operations [math knowledge] background with them, most of them, ... know addition and subtraction a little better, but no multiplication..., we tried to explain them a bit. I don’t even think they understand when they read a problem. [I don’t know] if they do not understand much because of the language ability of the children or because they don’t read much. We try to only focus on [basic] operations [with these children], ...This is also difficult. We don’t teach equations etc. to these 7th graders.

T5: Onlarla dört işlemler ile temeli güçlendirme adına ...Çoğu mesela toplama-çıkarmayı daha iyi biliyorlar ama çarpma yok… Onları biraz anlatmaya çalıştık. Hani en azından bir problemi okuduğunda anladığını pek düşünmüyorum. Çocukların da dil yapısından mı kaynaklanıyor, çok okumadıklarından mı anlayamıyorlar [bilmiyorum]. Sadece [bu çocuklarla] [dört] işlem odaklı çalışıyoruz, ...İşte bu da zor oluyor. 7’lerde falan denklem öyle şeyler öğretemiyoruz o çocuklara.”

As seen in the quote, T5 set lower expectations for the students with language barriers compared to the other students in face-to-face mathematics instruction. Since T5 could not facilitate synchronous lessons, due to students’ financial constraints and lack of access to the Internet, these students completely lost opportunities to learn mathematics during remote instruction.

## Discussion and conclusion

This study documented teachers’ perspectives on factors influencing equity in mathematics, namely, access to resources, teachers’ beliefs and decisions relative to high-quality mathematics instruction, and the support mechanisms offered that helped to extend student mathematical learning opportunities. The teachers’ perspectives identified that during remote instruction, most of the students who were disadvantaged economically, culturally, and linguistically experienced a wider learning gap due to a lack of access to the Internet and television. Also, students’ socioeconomic status and language barriers contributed to inequitable learning opportunities. Our findings aligned with researchers who acknowledged inequities within Turkish mathematics education settings (Baysu & Ağırdağ, 2019; MoNE, 2015; Özdemir, 2016; Tunga et al., 2020). Admittedly, our study findings showed that despite reform movements in Turkey, inequities during face-to-face instruction remain and are amplified in remote instruction due to COVID-19. Therefore, to empower teachers to attend to equity in mathematics teaching and learning, there is a need for intentional efforts to plan for and subsequently enact equitable practices within mathematics classes.

Our results aligned with other research findings (e.g., Çiftçi & Çağlar, 2014; Hohlfeld et al., 2017; OECD, 2019; Yetkiner Özel et al., 2013), which suggests that the socioeconomic status contributes to issues of access to mathematics and equitable learning opportunities. It also contributed to the extent that the students had access to technological devices such as computers, tablets, and televisions which played an inevitable role in accessing mathematics teaching and learning during remote instruction. Due to the constraints of lack of access, some of the teachers identified actions to overcome these challenges. Nevertheless, greater efforts are needed to provide access and equitable learning opportunities for all students to reduce learning gaps in mathematics. Thus, if intentional efforts are not systematically implemented, learning gaps in mathematics education would widen.

This study showed that teachers’ beliefs and expectations play an essential role in making curricular decisions to ensure equity for all students. Studies (Cross, 2009; NCTM, 2014; Philipp, 2007) have documented that teacher’s beliefs can influence their instruction and decision-making. Such studies also acknowledge that unproductive beliefs can negatively influence students’ learning opportunities (NCTM, 2014; Philipp, 2007). Similarly, this study found that setting high expectations for all students amplified the teachers’ efforts to support learning and create equitable mathematics learning opportunities during remote instruction. On the other hand, setting lower expectations hindered the extent teachers sought to address access issues and meet the learning needs of students who struggled academically or had language barriers. Thus, it is important to provide teachers with professional development opportunities about productive and unproductive beliefs (NCTM, 2014) and on how to implement, utilize, and possibly modify the support mechanisms that can meet students’ learning needs, particularly during remote instruction.

Furthermore, in this study, teachers acknowledged that students’ readiness to learn mathematical content is a factor that contributes to the extent that equity is attended to during face-to-face and remote modes of instruction. Not all students were able to benefit from synchronous instruction. Moreover, the teachers in this study were not involved in making curricular decisions to align the depth and pacing of mathematics contents in asynchronous video lessons broadcasted in EBA-TV according to their students’ mathematical readiness. These constraints led to an increase in the students’ mathematical knowledge gap during remote instruction. Therefore, educators, stakeholders, and policymakers can reflect on ways to improve mathematics instruction responsive to students’ readiness level during remote instruction to support equitable learning opportunities.

The teachers in this study acknowledged that cultural and language barriers contributed to widening the gap in students’ access to equitable mathematics instruction, which may negatively influence students’ mathematics learning. Thus, teachers are encouraged to exhibit culturally responsive mathematics teaching that capitalizes on students’ cultural funds of knowledge (Aguirre & Zavala, 2013; Gay, 2018). Also, to alleviate the cultural and linguistic problems, a more regional approach may be helpful in identifying the specific needs and problems of students in different regions. Additionally, providing resources about cultural nuances and professional support focusing on culturally-responsive mathematics teaching can facilitate equitable mathematics learning opportunities for all students.

The teachers participating in this study acknowledged that student engagement and interaction are not of the same quality in synchronous online lessons when compared to face-to-face instruction. In synchronous online lessons, the teachers had difficulties in using the strategies and support mechanisms that they were accustomed to during face-to-face instruction to enable student engagement and to elicit students’ mathematical thinking. Thus, it is suggested that the teachers should be supported on how to adapt and revise their existing strategies for enabling interaction and engagement in remote instruction.

The perspective of teachers on equity and accessibility suggested that there are many challenges that influence equitable mathematics instruction practices in remote instruction. Some of these challenges included language barriers and teachers’ expectations and beliefs. These challenges are difficult to tackle because it is not merely a matter of providing access or resources but addressing perspectives that are influenced by cultural norms and lived experiences. It is possible to overcome these challenges with a multi-step approach that empowers teachers to enact strategies for rigorous mathematics learning regardless of the contextual setting. Documenting teachers’ perspectives on the factors that support or hinder equity could give an opportunity for educators and policymakers to notice the improvement areas for creating equitable remote mathematics instruction.

Although the teachers in this study acknowledged inequities during remote instruction, when they were asked about issues of equity in mathematics, the majority of them provided general responses and struggled to contextualize it for mathematics. This general description of equity might be related to teachers’ abilities to notice equitable practices in a mathematics classroom (Erickson, 2011; Hand et al., 2012; van Es et al., 2017; Wager, 2014). As van Es et al. (2017) suggested, teachers are inclined to describe what happens in their classrooms in general terms instead of noticing the nuanced differences among diverse student groups in relation to their instructional decision. Similarly, in this study, teachers acknowledged the limitations of student engagement and interaction in online lessons as an example. Yet they did not share their perspectives on the details regarding how different student groups interact around mathematical ideas (Wager, 2014). Another finding from this study was that teachers tended to consider their students’ individual characteristics (e.g., being second language learners, culture, socioeconomic status) when they speak to equity in mathematics. However, only a few teachers gave mathematics-specific responses emphasizing the importance of providing mathematics learning opportunity that draws on students’ experiences and culture. Thus, the scarcity of studies and professional support on equity in mathematics for teachers in Turkey and the infancy of the studies on teacher noticing on equitable mathematics practice (van Es et al., 2017) could be the important reasons why the teachers in this study could not contextualize their responses for mathematics. However, we also suggest that further research should be conducted to investigate why teachers have challenges in contextualizing their responses for mathematics.

We acknowledge that the small sample size of our study is a limitation and is not generalizable. Nevertheless, the finding provides an insight into the challenges to attend to equity in mathematics during remote instruction, within the Turkish setting. Additionally, it is important to note that after the study was conducted, ongoing revisions were being made for remote instruction in Turkey. Some of the revisions made sought to address a few of the challenges of inequities in mathematics such as providing support for mathematically struggling students with the Remedial Education Program in Primary Schools (MoNE, 2018c) on EBA-TV (https://www.eba.gov.tr/arama?q=iyep). Thus, potentially positive changes are happening to support students’ learning. Therefore, future studies can examine the nature of mathematics instructional support offered in subsequent years and on the impact of professional development on equity in mathematics.

## Footnotes

1 Lesson videos can be accessed at https://www.trtizle.com/uzaktan-egitim.

## Declarations

University of California Riverside Institutional Review Board approved the study with reference number HS-20-111. Informed consents from all participants were received electronically. All authors affirm that this manuscript is an honest, accurate, and transparent account of the study being reported that no important aspects of the study have been omitted.

### Disclaimer

All authors declare that this manuscript is original, has not been published before and is not currently being considered for publication elsewhere.
